# Supplementary material for: The interactome of intact mitochondria by cross-linking mass spectrometry provides evidence for coexisting respiratory supercomplexes
Source: Mol Cell Proteomics. 2017 Dec 8;17(2):216–32. doi: 10.1074/mcp.RA117.000470 (PMC5795388; doi:10.1074/mcp.RA117.000470)
Supplement: Supplemental Data [file supp_RA117.000470_133922_0_supp_23978_fzffwf.zip › spectra_annotation/mito_DR_spectra_annotation/285-1-4-1-22-1.pdf]

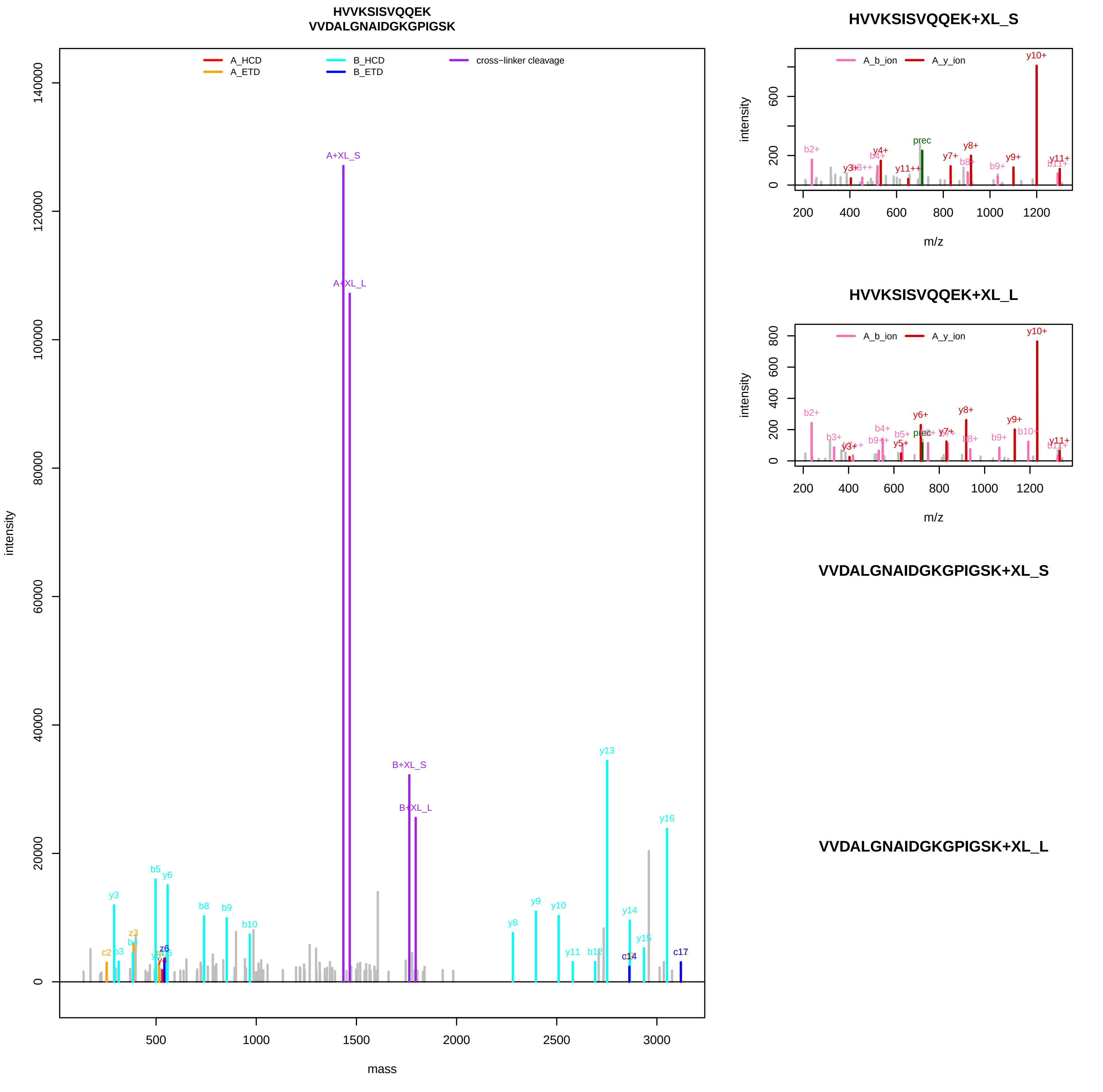
HVVKSISVQQEK
VVDALGNAIDGKGPIGSK
A_HCD
A_ETD
B_HCD
B_ETD
cross−linker cleavage
intensity
mass
A+XL_S
A+XL_L
B+XL_S
B+XL_L
y3
b5
y6
b8
b9
b10
c2
z3
z6
y8
y9
y10
y11
b12
y13
y14
y15
y16
c14
c17
HVVKSISVQQEK+XL_S
A_b_ion
A_y_ion
m/z
prec
b2+
y3+
y4+
b4+
y11++
y7+
y8+
b8+
b9+
y9+
y10+
y11+
b11+
HVVKSISVQQEK+XL_L
b3+
y5+
b5+
y6+
b10+
VVDALGNAIDGKGPIGSK+XL_S
VVDALGNAIDGKGPIGSK+XL_L
